# Supplementary material for: Wood-Decaying Fungi: From Timber Degradation to Sustainable Insulating Biomaterials Production
Source: Materials (Basel). 2023 May 5;16(9):3547. doi: 10.3390/ma16093547 (PMC10179824; doi:10.3390/ma16093547)
Supplement: Supplementary file 1 [file materials-16-03547-s001.zip › materials-2333309-supplementary.pdf]

Supplementary S1

Two Way Analysis of Variance (without replication) for Morphometric parameters, obtained by X-ray micro-CT, of the nine bio-material specimens.

Detailed ANOVA results and all pairwise comparisons regarding only the three parameters for which factors "Substrate" or "Fungus" (or both) produce significant effects are reported here. For all the other parameters any significant effect of "Substrate" or "Fungus" factor has resulted.

- Dependent Variable: **Mean pore size**

Normality Test (Shapiro-Wilk): Passed (P = 0,973)
Equal Variance Test (Brown-Forsythe): Passed (P = 1,000)

Source of Variation	DF	SS	MS	F	P
Substrate	2	177601,197	88800,598	9,969	0,028
Fungus	2	49298,616	24649,308	2,767	0,176
Residual	4	35631,622	8907,906		
Total	8	262531,436	32816,429		

The difference in the mean values among the different levels of Substrate is greater than would be expected by chance after allowing for effects of differences in Fungus. There is a statistically significant difference (P = 0,028). To isolate which group(s) differ from the others use a multiple comparison procedure.

The difference in the mean values among the different levels of Fungus is not great enough to exclude the possibility that the difference is just due to random sampling variability after allowing for the effects of differences in Substrate. There is not a statistically significant difference (P = 0,176).

Power of performed test with alpha = 0,0500: for Substrate : 0,709
Power of performed test with alpha = 0,0500: for Fungus : 0,200

Least square means for Substrate :

Group	Mean
Ailanthus	421,760
Straw	422,076
Sawdust	123,924

Std Err of LS Mean = 54,491

Least square means for Fungus :

Group	Mean
Ganoderma l	317,875
Pleurotus o	415,495
Trametes v	234,390

Std Err of LS Mean = 54,491

All Pairwise Multiple *Comparison Procedures (Tukey Test):
Comparisons for factor: **Substrate**

Comparison	Diff of Means	p	q	P	P<0,050
Straw vs. Sawdust	298,152		35,472	0,039	Yes
Straw vs. Ailanthus	0,316		30,00581	1,000	No
Ailanthus vs. Sawdust	297,836		35,466	0,039	Yes

Comparisons for factor: **Fungus**

Comparison	Diff of Means	p	q	P	P<0,050
Pleurotus o vs. Trametes v	181,105		33,324	0,159	No
Pleurotus o vs. Ganoderma l	97,620		31,791	0,482	Do Not Test
Ganoderma l vs. Trametes v	83,485		31,532	0,572	Do Not Test

- Dependent Variable: **Specimen specific surface**

Normality Test (Shapiro-Wilk): Passed (P = 0,796)
Equal Variance Test (Brown-Forsythe): Passed (P = 1,000)

**Source of Variation**	**DF**	**SS**	**MS**	**F**	**P**
Substrate	2	43530,769	21765,384	30,371	0,004
Fungus	2	4736,945	2368,473	3,305	0,142
Residual	4	2866,612	716,653		
Total	8	51134,326	6391,791		

The difference in the mean values among the different levels of Substrate is greater than would be expected by chance after allowing for effects of differences in Fungus. There is a statistically significant difference (P = 0,004). To isolate which group(s) differ from the others use a multiple comparison procedure.

The difference in the mean values among the different levels of Fungus is not great enough to exclude the possibility that the difference is just due to random sampling variability after allowing for the effects of differences in Substrate. There is not a statistically significant difference (P = 0,142).

Power of performed test with alpha = 0,0500: for Substrate : 0,993
Power of performed test with alpha = 0,0500: for Fungus : 0,247

Least square means for Substrate :

**Group**	**Mean**
Ailanthus	192,859
Straw	147,444
Sawdust	312,343

Std Err of LS Mean = 15,456

Least square means for Fungus :

**Group**	**Mean**
Ganoderma l	209,963
Pleurotus o	194,023
Trametes v	248,661

Std Err of LS Mean = 15,456

All Pairwise Multiple *Comparison Procedures (Tukey Test):
Comparisons for factor: **Substrate**

**Comparison**	**Diff of Means**	**p**	**q**	**P**	**P<0,050**
Sawdust vs. Straw	164,899	3	10,669	0,004	Yes
Sawdust vs. Ailanthus	119,484	3	7,731	0,012	Yes
Ailanthus vs. Straw	45,415	3	2,938	0,210	No

Comparisons for factor: **Fungus**

**Comparison**	**Diff of Means**	**p**	**q**	**P**	**P<0,050**
Trametes v vs. Pleurotus o	54,638	3	3,535	0,136	No
Trametes v vs. Ganoderma l	38,698	3	2,504	0,289	Do Not Test
Ganoderma l vs. Pleurotus o	15,940	3	1,031	0,761	Do Not Test

- Dependent Variable: **Fractal dimension**

Normality Test (Shapiro-Wilk): Passed (P = 0,825)
Equal Variance Test (Brown-Forsythe): Passed (P = 1,000)

Source of Variation	DF	SS	MS	F	P
Substrate	2	0,0588	0,0294	23,898	0,006
Fungus	2	0,0136	0,00681	5,533	0,070
Residual	4	0,00492	0,00123		
Total	8	0,0773	0,00967		

The difference in the mean values among the different levels of Substrate is greater than would be expected by chance after allowing for effects of differences in Fungus. There is a statistically significant difference (P = 0,006). To isolate which group(s) differ from the others use a multiple comparison procedure.

The difference in the mean values among the different levels of Fungus is not great enough to exclude the possibility that the difference is just due to random sampling variability after allowing for the effects of differences in Substrate. There is not a statistically significant difference (P = 0,070).

Power of performed test with alpha = 0,0500: for Substrate : 0,976
Power of performed test with alpha = 0,0500: for Fungus : 0,433

Least square means for Substrate :

Group	Mean
Ailanthus	2,720
Straw	2,735
Sawdust	2,899

Std Err of LS Mean = 0,0203

Least square means for Fungus :

Group	Mean
Ganoderma l	2,757
Pleurotus o	2,758
Trametes v	2,840

Std Err of LS Mean = 0,0203

All Pairwise Multiple *Comparison Procedures (Tukey Test):
Comparisons for factor: **Substrate**

Comparison	Diff of Means	p	q	P	P<0,050
Sawdust vs. Ailanthus	0,178	3	8,806	0,008	Yes
Sawdust vs. Straw	0,164	3	8,082	0,010	Yes
Straw vs. Ailanthus	0,0147	3	0,724	0,870	No

Comparisons for factor: **Fungus**

Comparison	Diff of Means	p	q	P	P<0,050
Trametes v vs. Ganoderma l	0,0830	3	4,099	0,092	No
Trametes v vs. Pleurotus o	0,0820	3	4,049	0,095	Do Not Test
Pleurotus o vs. Ganoderma l	0,00100	3	0,0494	0,999	Do Not Test

* A result of "Do Not Test" occurs for a comparison when no significant difference is found between two means that enclose that comparison. For example, if you had four means sorted in order, and found no difference between means 4 vs. 2, then you would not test 4 vs. 3 and 3 vs. 2, but still test 4 vs. 1 and 3 vs. 1 (4 vs. 3 and 3 vs. 2 are enclosed by 4 vs. 2: 4 3 2 1). Note that not testing the enclosed means is a procedural rule, and a result of Do Not Test should be treated as if there is no significant difference between the means, even though one may appear to exist.
